# Digestible Energy Intake and Digestive Efficiency of Human-Managed North American River Otters (*Lontra canadensis*)

**DOI:** 10.1155/2020/4307456

**Published:** 2020-01-20

**Authors:** Larry J. Minter, Kimberly Ange-van Heugten, Craig A. Harms, Michael K. Stoskopf

**Affiliations:** ^1^Hanes Veterinary Medical Center, North Carolina Zoo, 4401 Zoo Parkway, Asheboro, NC 27205, USA; ^2^Department of Clinical Sciences, North Carolina State University, College of Veterinary Medicine, 1060 William Moore Drive, Raleigh, NC 27607, USA; ^3^Environmental Medicine Consortium, North Carolina State University, 1060 William Moore Drive, Raleigh, NC 27607, USA; ^4^Department of Animal Sciences, North Carolina State University, Box 7621, Raleigh, NC 27695, USA; ^5^Department of Clinical Sciences, Center for Marine Sciences and Technology, North Carolina State University, College of Veterinary Medicine, 303 College Circle, Morehead City, NC 28557, USA

## Abstract

Diets currently provided to captive North American river otters (*Lontra canadensis*) are highly variable with different institutions providing various whole foods, commercial complete prepared diets, or combinations of both. This study investigated the digestible energy intake, gastrointestinal transit time, and digestive efficiency of three different diets being fed at three North Carolina institutions. Otters housed at Institution A (*n* = 3) were fed strictly fish. Otters housed at Institutions B (*n* = 3) and C (*n* = 2) were fed a majority fish based diet (58.5 and 74.1%, respectively), supplemented with fruits, vegetables, and supplemental protein sources as enrichment. There was an apparent trend between increased percentage of fish in the diet and faster transit time and higher digestive efficiency. As less fish was included in the diets, the GI transit time was longer (Institution A, 106 minutes; Institution B, 145 minutes; Institution C, 208 minutes). Median digestive efficiency was high for all three groups (A, 91.4%; B, 87.8%; C, 89.8%) but was higher for the institutions feeding fish. Additionally, the overall median gross energy intake for the eight animals in this study was 163.1 kcal/kgBM^0.75^/day (range: 92.2 to 260.7 kcal/kgBM^0.75^/day). While all three institutions had healthy otter populations, it appears that a higher fish diet should be further studied as the model North American river otter diet.

## 1. Introduction

The North American river otter (*Lontra canadensis*) is a semiaquatic opportunistic predator that feeds primarily on fish but also a wide variety of other prey items (e.g., crustaceans, amphibians, reptiles, birds, mammals, and insects) [1–6]. Once found in virtually every watershed in North America, they are being successfully reestablished in many areas after being extirpated from much of their original range [2, 7, 8].

The success of human-managed populations of North American river otters depends on proper nutrition [9, 10]. The diets currently provided to human-managed North American river otters vary considerably. Some institutions provide a variety of whole food items (e.g., fish, shellfish, rodents, fruits, vegetables, and chicken), others feed commercial nutritionally complete pre-prepared diets, while few provide a mixture of both [11, 12]. While the digestibility of different nutrients in commercial diets has been described and documented for domestic dogs and cats, comprehensive dietary evaluations for most managed zoological carnivores are limited [10, 13–16].

The objective of this study was to determine the gross energy intake, gastrointestinal transit time, and apparent digestibility of gross energy, crude protein, and crude fat of three different diets being fed to three different managed North American river otter populations.

## 2. Materials and Methods

### 2.1. Study Animals

Eight adult North American river otters held in three North Carolina institutions denoted in this paper as Institution A, *n* = 3 (1 male; 2 females); Institution B, *n* = 3 (3 males); and Institution C, *n* = 2 (1 male; 1 female) (Table 1), were used in this study. All of the animals appeared outwardly healthy, based on routine clinical examinations. One of the animals housed at Institution B was considered moderately overconditioned (7/9) based on the clinical body condition score (1 to 9 scale with 1 being very thin, 9 being grossly obese, and 5 being ideal), while all of the other animals in this study were considered close to an ideal body condition (5 to 6/9). All procedures in this study were reviewed and approved by the appropriate research review committees for each of the participating institutions. Except for an individually housed female at Institution A, animals were group housed at each institution. The animals exhibited at Institutions B and C were housed completely indoors with exposure to natural lighting via skylights, while the enclosure at Institution A was outdoors with indoor holding. For the duration of this study, all animals were isolated during feeding and then returned to their groups.

### 2.2. Animal Diets

Otters housed at Institution A were fed a fish-only diet. Otters housed at Institutions B and C were fed different fish-based diets (Institution B, 58.5% fish; Institution C, 74.1% fish) supplemented with fruits and vegetables along with supplemental protein sources (e.g., krill, crayfish claws, shrimp, and hard boiled eggs) (Table 2). All animals were fed twice daily as follows (wet mass): Institution A, 659.0, 751.2, and 914.2 g/animal/day; Institution B, 898.8, 1008.12, and 1087.4 g/animal/day; and Institution C, 507.4 and 575.5 g/animal/day, and provided a thiamine and vitamin E supplement (Institution A: Mazuri Thiamin-E Paste, Henderson, CO; Institution B: Mazuri Marine Mammal Tablet, Henderson, CO; Institution C: VPL Optima 365, Phoenix, AZ). Water was provided ad libitum.

### 2.3. Nutritional Analysis

Gastrointestinal transit time and total fecal collection digestibility assessments were performed over a 3-day period. Uniquely colored markers (McCormick & Company, Inc., Sparks Glencoe, MD) were added to each diet to identify individual otter scat in the group-housing situations. The rate of food transit was measured on Day 1 of the diet trial. The time of ingestion of the marked diet was recorded, and the animal was observed every 2½ minutes until the appearance of the marked feces. Gastrointestinal transit time was calculated as the interval between the first ingestion of the diet and the appearance of marked feces.

Prior to each feeding on Days 1 and 2, the wet weight of each diet component was recorded. All of the food left uneaten (ort) was immediately collected and weighed. Total feces were collected throughout the day and the following mornings and weighed. Four separate samples of each fish species fed, each of the supplemental enrichment portions of the diets, and feces from individual animals were individually homogenized in a kitchen blender (Oster, Inc., Neosho, MO), and the homogenates were placed in an ultralow freezer (Thermo Fisher Scientific, Inc., Waltham, MA) set at −70°C and stored up to 4 months until analyzed.

To determine percent dry matter, aliquots of frozen samples were thawed and then dried under forced air at 60°C for 72 hrs. To determine adequate drying, repetitive weights were acquired after each drying, and when weight change between weighings was less than 0.1 g, a sample was considered dry matter. Percent dry matter was calculated by dividing dry weight by wet weight multiplied by 100 (AOAC method 950.46). Dried samples were ground using a handheld coffee grinder (Krups, Inc., Millville, NJ), and gross energy was determined for each fish species fed, the supplemental diet enrichment component, and each fecal sample in duplicate using an IKA C5000 bomb calorimeter (IKA® Works, Inc., Wilmington, NC). Duplicate subsamples were averaged to yield a single data point. Variation in duplicate subsamples was less than 4.0% for all diet items and feces samples analyzed. Fish, supplemental diet enrichment, and feces were analyzed for crude fat by Soxhlet ether extraction (AOAC method 2003.05) and crude protein using a Leco nitrogen/protein determinator (Leco FP-528, LECO Corporation, St. Joseph, MI; AOAC method 992.15) in duplicate by a commercial laboratory service (Zooquarius, Inc., Ithaca, NY). All nutritional compositions were calculated and presented on a dry matter basis (DMB). Percent apparent nutrient digestibility was then calculated using the following formula [17]:

(1) $$\begin{array}{c} \text{apparent nutrient digestibility}\left(\%\right)=\frac{\text{nutrient intake}-\text{nutrient in feces}}{\text{nutrient intake}}\times 100\%. \end{array}$$

### 2.4. Statistical Analysis

Descriptive statistics were calculated using JMP Pro, version 11.0 software (SAS Institute Inc., Cary, NC). The data for each variable were tested for normality of distribution by the Shapiro–Wilk test. Summary statistics are presented as both median and ranges.

## 3. Results

The nutrient composition of the commercially available fish used in the diets varied (Table 3). Gross energy (DMB) ranged from 5136 to 6955 cal/g across the species of fish sampled. Whole catfish was the fattiest, with a crude fat of 56.0%, and had the lowest protein with a crude protein of 39.2%. *Tilapia* and salmon fillets used in some diets had the lowest crude fat (9.9% and 9.3%, respectively) and the highest crude protein (89.0% and 88.6%, respectively) of all fish species tested.

Gross energy for each of the actual overall diets fed to the animals was within 10% of each other (range: 5272 to 5622 cal/g; Table 4). The crude protein of overall diets varied more widely (range: 53.7 to 73.6%; Table 3) and was nearly 35% higher for the diet composed of entirely fish compared to the two diets composed of fish supplemented with fruits and vegetables. The fat composition of the Institution C diet (29.9%) was similar to the crude fat of the Institution B diet (23.4%), but the diet for Institution A (entirely fish) contained just over half the amount of crude fat (15.7%) of diets fed at Institutions B and C (fish supplemented with fruits and vegetables).

The median gross energy intake for all eight animals was 163.1 kcal/kgBM^0.75^/day (range: 92.2 to 260.7 kcal/kgBM^0.75^/day). Median gross energy was the highest for otters held at Institution B (228.5 kcal/kgBM^0.75^/day), followed by Institution A (154.5 kcal/kgBM^0.75^/day) and Institution C (144.2 kcal/kgBM^0.75^/day; Table 5).

The gastrointestinal transit time for animals fed only fish tended to be faster than that for animals fed fish along with vegetables and fruits (Institution A, 106 minutes; Institution B, 145 minutes; Institution C, 208 minutes; Table 6).

Apparent digestibility of gross energy, crude protein, and crude fat differed only slightly between the three institutions (ranges: gross energy, 87.8 to 91.4%; crude protein, 87.8 to 92.1%; crude fat, 90.4 to 96.5%; Table 6). Apparent digestibility for gross energy, crude protein, and crude fat was highest for those animals fed only fish diet (Institution A).

## 4. Discussion

The crude nutrient compositions for the commercially available fish species analyzed in this study were in the same range as previously published values [18, 19]. While whole fish is a good source of most nutrients, the nutrient parameters vary a great deal among fish species, and within fish species based on diet, season, and lifecycle/physiological stage of the fish [18–20]. The values for the nutrient parameters presented here provide only a snap shot of the varying nutrient composition of fish provided to these animals over time. Basic diet analysis such as gross energy, crude fat, crude protein, vitamins, and minerals should be performed with every lot of fish being used in the diet preparation for otters. This ensures that the animals are being provided a balanced diet, as seasonal changes in these nutrient parameters can lead to a large inconsistency in the intake gross energy, crude fat, and crude protein with similar quantities of fish being consumed [19].

The nutritional needs of the North American river otters are often extrapolated from the requirements of domestic cats and diets that have been successfully used to maintain captive otters [12, 14]. The gross energy found in the three primarily fish-based diets of this study was similar to those reported for other primarily teleost fish diets fed to harp seals (*Phoca groenlandica*) (range: 4680 to 6690 cal/g), Hawaiian monk seal (*Monachus schauinslandi*) (range: 4760 to 5740 cal/g), and Steller sea lion (*Eumetopias jubatus*) (6001 cal/g) as well as several commercially prepared diets (polar bear, exotic feline, and domestic cat) (range: 4720 to 6690 cal/g) fed North American river otters [11, 21–23]. The high gross energy needs suggested for North American river otters and provided in the diets of this study address a concern for a high energy demand for this species. This concern for a high energy demand is based on the high metabolic rate for this species, which is approximated at 20 to 48% greater than that expected for most other mammals [24, 25].

Protein, carbohydrate, and fat all provide energy in a diet, with fat providing energy on average twice that of protein and carbohydrates [12]. The fish-only diet provided to the otters at Institution A contained the highest concentration of protein of the three diets analyzed, but the crude protein concentration of all three diets was well above the minimum recommendations reported in the North American River Otter Husbandry Manual (24 to 32.5% crude protein) and similar to that of several diets being fed to both captive North American river otters (33.3 to 54.1% crude protein) and Asian small-clawed otters (42.9 to 68.1% crude protein) [11, 12, 26]. Crude fat concentration in the three diets analyzed for this study was consistent with the current minimum recommendation for the North American river otters (15 to 30% crude fat) and that of numerous diets being fed to captive otter species (11.3 to 37.9% crude fat) [11, 12, 26]. Crude fat concentration varies more than 50% in diets being fed to otter species with no reported clinical signs of steatorrhea, pancreatitis, or significant pathological changes in the cardiovascular system suggesting that otters have the capacity to tolerate and utilize variable levels of dietary fats [11, 12, 26].

The median gross energy intake was numerically higher for the otters housed at Institution B when evaluated against the animals at either Institution A or C. This was expected because the otters at Institution B were fed a greater amount of diet by wet weight when compared to the animals in the other institutions. The median gross energy intake established from the otters in this study was consistent with reports on gross energy intake for both North American otters (177.0 kcal/kgBM^0.75^/day) and Eurasian river otters (*Lutra lutra*) (171.9 and 225.5 kcal/kgBM^0.75^/day) and fishers (*Martes pennanti*) (131.4 to 188.8 kcal/kgBM^0.75^/day) fed several different diets comprising various whole food items and slightly higher than those reported for other comparable sized terrestrial mammals (domestic cat (*Felis silvestris catus*), 42.0–134.7 kcal/kgBM^0.75^/day; bobcat (*Lynx rufus*), 46.3 kcal/kgBM^0.75^/day; African wildcats (*Felis silvestris lybica*), 91.5 to 120.5 kcal/kgBM^0.75^/day) [27–35].

The gastrointestinal transit time of the North American river otter is much faster than that reported for many other terrestrial carnivores [36]. This would presumably allow less time for complete hydrolysis and absorption of nutrients. The otters fed a strictly fish diet in this study had a notably faster transit time than the otters fed fish supplemented with both fruits and vegetables. The median transit time for the otters at Institution A was also faster than previous data from North American otters fed a diet of various whole food items (202 minutes) and those fed several different commercially available diets (167 to 188 minutes) [11, 30]. Diets with a higher percentage of crude fat (Institutions B and C) appeared to slow down the passage of food through the gastrointestinal system in the otters in this study. The increasing fat content of a diet reportedly delays stomach emptying, thereby decreasing the passage rate of food [37, 38]. This trend was also observed in North American river otters fed several different commercially available diets with varying degrees of crude fat [11]. The addition of both insoluble and soluble fibers from the supplementation of both fruits and vegetables may also have affected the transit time of the animals in this study though regrettably analysis of the total dietary fiber was not performed.

The median apparent digestibility of gross energy for all three groups in this study was consistent with reports measuring apparent digestibility of gross energy in North American river otters fed Nebraska Feline Diet (90.2%) (North Platte, NE), Mazuri Polar Bear Diet (83.1%) (Henderson, CO), and Hill's Science Diet cat food (86.6%) (Topeka, KS), as well as fishers fed several different diets comprising various whole food items (80.9 to 92.6%) and both walrus (*Odobenus rosmarus*) (92.7%) and Steller sea lions fed a diet of herring (95.5%) [11, 23, 27, 39]. The animals in this study fed strictly fish appeared to be slightly more efficient at the digestion of gross energy, crude fat, and crude protein than those animals supplemented with both fruits and vegetables. The decrease in apparent digestibility of nutrients (gross energy, crude fat, and crude protein) in the animals at both Institutions B and C could be related in part to the supplementation of fruits and vegetables and therefore an increase in fibers or the less digestible fat in these food items. Our results were consistent with findings that an increase in fibers in the diets of mink (*Mustela vison*), blue fox (*Alopex lagopus*), and both the domestic cat and dog decreased the apparent digestibility of crude protein, which corresponded with the increase in concentration of plant protein, which is less likely digestible than the animal proteins it replaced [40–42]. It was anticipated that the higher concentration of crude fat in the diets at both Institutions B and C would have increased the apparent digestibility for the nutrients analyzed (gross energy, crude fat, and crude protein) for those diets similar to what has been reported for dogs and blue foxes with the addition of fat to their diets [43, 44]. That was not the case in our study. While not measured in our study, the addition of fibers to the diets through the supplementation of fruits and vegetables may explain the increase in the excretion of fecal fat, thereby decreasing the apparent digestibility of crude fat [45].

Digestibility trials can be difficult to perform on animals housed in more natural habitats in zoological collections due to the difficulty of conducting total feces collection. This difficulty can be overcome by comparing the changes in concentrations of an inert dietary marker [17]. The animals in this study used the same latrine sites which allowed us to collect a complete fecal sample making dietary markers unnecessary to determine apparent digestibility.

This study indicated an apparent trend between an increase in the percentage of fish offered in the diet and a faster transit time and higher digestive efficiency. Knowledge on energy content, gastrointestinal transit time, and apparent digestibility of nutrients associated with a more fish-based diet is important if zoological institutions are to provide a nutrient dense diet sufficient for maintenance, growth, and reproduction in this highly active species. This study did not examine the digestibility of any of the diets in regard to vitamins and minerals, and further studies should be performed to evaluate these nutrient parameters and to ensure that these animals are being provided a completely balanced diet.

## Figures and Tables

**Table 1:** Sex, age, and body mass (kg) of the North American river otters (*Lontra canadensis*) held at three North Carolina institutions.

Age class	Sex	Age (yrs)	Body mass (kg)	Institution
Adult	M	6	7.9	A
Adult	F	10	7.7	A
Adult	F	16	7.6	A
Adult	M	4	7.6	B
Adult	M	7	7.8	B
Adult	M	7	9.6	B
Adult	M	15	7.8	C
Adult	F	11	6.9	C

**Table 2:** Percent distribution of food items in the diets of the captive North American river otters (*Lontra canadensis*) held at three North Carolina institutions (weight as fed).

% Institution A^a^		% Institution B^a^		% Institution C^a^	
Mackerel (whole)	47.2	Smelt (whole)	18.7	Catfish (whole)	37.6
Smelt (whole)	28.3	Trout (whole)	15.4	Capelin (whole)	36.5
Capelin (whole)	24.5	Perch (fillet)	8.5	Enrichment items^c^	25.9
		Tilapia (fillet)	6.8		
		Mullet (whole)	5.6		
		Salmon (fillet)	3.5		
		Enrichment items^b^	41.5		

^a^Each diet was supplemented with thiamine and vitamin E. ^b^Cantaloupe, carrot, cauliflower, celery, green pepper, mango, mushroom, papaya, sugar pea, sweet potato, tomato, and whole peanuts. ^c^Banana, blackberry, blueberry, broccoli, carrot, crayfish claw, hardboiled egg, krill, mushroom, nectarine, peanut butter, pear, sweet potato, whole clam, and whole shrimp with shell.

**Table 3:** Nutrient content (dry matter basis) of the commercially available fish fed in the diet trials for eight captive North American river otters (*Lontra canadensis*) held at three North Carolina institutions.

Diet component	DM (%)	Gross energy (cal/g)	Crude protein (%)	Crude fat (%)
Capelin (whole)	12.15	5136	77.1	11.3
Catfish (whole)	18.29	6955	39.2	56.0
Mackerel (whole)	24.71	5526	74.2	15.1
Mullet (whole)	16.36	5481	58.6	28.9
Perch (fillet)	3.31	5166	74.5	14.2
Salmon (fillet)	12.05	5400	88.6	9.3
Smelt (whole)	8.65	5830	69.5	20.6
Tilapia (fillet)	9.36	5468	89.0	9.9
Trout (whole)	12.87	5865	60.8	27.9

**Table 4:** Nutrient content (dry matter basis) of complete diets used in diet trials for eight captive North American river otters (*Lontra canadensis*) held at three North Carolina institutions.

Institution	Gross energy (cal/g)	Crude protein (%)	Crude fat (%)
Institution A (*n* = 3)	5516	73.6	15.7

Institution B (*n* = 3)	5272	53.8	23.4

Institution C (*n* = 2)	5622	53.7	29.9

Data are reported as median values.

**Table 5:** Daily intake of gross energy in eight captive North American river otters (*Lontra canadensis*) held at three North Carolina institutions.

Institution	Gross energy intake (kcal/day)		Gross energy intake (kcal/kgBM^0.75^/day)	
	Median	Range	Median	Range
Institution A (*n* = 3)	645.3	435.5–793.8	154.5	92.2–171.7
Institution B (*n* = 3)	1073.2	949.4–1195.9	228.5	173.5–260.7
Institution C (*n* = 2)	645.1	600.5–689.8	144.2	141.4–146.9

**Table 6:** Nutrient digestive efficiency and gastrointestinal transit time for eight captive North American river otters (*Lontra canadensis*) held at three North Carolina institutions.

Institution	Gross energy digestibility		Crude protein digestibility		Crude fat digestibility		GI transit time	
	Median (%)	Range (%)	Median (%)	Range (%)	Median (%)	Range (%)	Median (min)	Range (min)
Institution A (*n* = 3)	91.4	91.3–92.5	92.1	91.26–92.8	96.5	95.7–97.2	106	98–114
Institution B (*n* = 3)	87.8	74.8–90.9	87.9	79.9–92.1	91.9	88.2–99.1	145	138–174
Institution C (*n* = 2)	89.8	88.2–91.4	87.8	85.8–89.8	90.4	89.9–90.8	208	207–209

## Data Availability

The full data set which was used for this research is available from the corresponding author on reasonable request.

## References

[1] Toweill D. E. (1974). Winter food habits of river otters in western Oregon. *The Journal of Wildlife Management*.

[2] Toweill D. E., Tabor J. E., Chapman J. A., Feldhamer G. A. (1982). The northern river otter *Lutra canadensis* (Schreber). *Wild Mammals of North America*.

[3] Larsen D. N. (1984). Feeding habits of river otters in coastal southeastern Alaska. *The Journal of Wildlife Management*.

[4] Tumlison R., Karnes M. (1987). Seasonal changes in food habits of river otters in southwestern Arkansas beaver swamps. *Mammalia*.

[5] Serfass T. L., Rymon L. M., Brooks R. P. (1990). Feeding relationships of river otters in northeastern Pennsylvania. *Transaction of Northeast Section of Wildlife Society*.

[6] Reid D. G., Code T. E., Reid A. C. H., Herrero S. M. (1994). Food habits of the river otter in a boreal ecosystem. *Canadian Journal of Zoology*.

[7] Melquist W. E., Dronkert A. E., Novak M., Baker J. A., Obbard M. E., Malloch B. (1987). River otter. *Wild Furbearer Management and Conservation North America*.

[8] Lariviere S., Walton L. R. (1998). Lontra felina. *Mammalian Species*.

[9] Dierenfeld E. S. (1997). Captive wild animal nutrition: a historical perspective. *Proceedings of the Nutrition Society*.

[10] Hatt J. M., Nijboer J., Hatt J. M., Kaumanns W., Bejnen A., Ganslober U. (2000). Nutrition research in zoo animals. *Zoo Animal Nutrition*.

[11] White S. C., Clark D. W., Day C. D., Sikes R. S. (2007). Variation in digestive efficiency of captive North American river otters (*Lontra canadensis*) on various diets. *Zoo Biology*.

[12] Reed-Smith J. (2012). *North American River Otter (Lontra canadensis): Husbandry Notebook, Section 2 Chapter 7–10*.

[13] Oftedal O. T., Allen M. E., Kleiman D. G., Allen M. E., Thompson K. V., Lumpkin S. (1998). Nutrition and dietary evolution in zoos. *Wild Mammals in Captivity – Principles and Techniques*.

[14] National Research Council (2006). *Nutrient Requirements of Dogs and Cats*.

[15] Vester B. M., Burke S. L., Dikeman C. L., Simmons L. G., Swanson K. S. (2008). Nutrient digestibility and fecal characteristics are different among captive exotic felids fed a beef-based raw diet. *Zoo Biology*.

[16] Clauss M., Kleffner H., Kienzle E. (2010). Carnivorous mammals: nutrient digestibility and energy evaluation. *Zoo Biology*.

[17] Khan M. A., Nisa M., Sarwar M. (2003). Techniques measuring digestibility for the nutritional evaluation of feeds. *International Journal of Agriculture and Biology*.

[18] Lawson J., Magalhães A., Miller E. (1998). Important prey species of marine vertebrate predators in the northwest Atlantic:proximate composition and energy density. *Marine Ecology Progress Series*.

[19] Bernard J. B., Allen M. A. (2002). *Feeding Captive Piscivorous Animals: Nutritional Aspects of Fish as Food*.

[20] Martensson P. E., Lager Gotass A. R., Norday E. S., Blix A. S. (1996). Seasonal changes in energy density of prey of northeast Atlantic seals and whales. *Marine Mammal Science*.

[21] Lawson J. W., Miller E. H., Noseworthy E. (1997). Variation in assimilation efficiency and digestive efficiency of captive harp seals (*Phoca groenlandica*) on different diets. *Canadian Journal of Zoology*.

[22] Goodman-Lowe G. D., Carpenter J. R., Atkinson S. (1999). Assimilation efficiency of prey in the Hawaiian monk seal (*Monachus schauinslandi*). *Canadian Journal of Zoology*.

[23] Rosen D. A., Trites A. W. (2000). Digestive efficiency and dry-matter digestibility in Steller sea lions fed herring, pollock, squid, and salmon. *Canadian Journal of Zoology*.

[24] Iversen J. A. (1972). Basal energy metabolism of mustelids. *Journal of Comparative Physiology*.

[25] Pfeiffer P., Culik B. M. (1998). Energy metabolism of underwater swimming in river-otters (*Lutra lutra L.*). *Journal of Comparative Physiology B: Biochemical, Systemic, and Environmental Physiology*.

[26] Maslanka M. T., Crissey S. D., Lombardi D., O’Connor J. (1998). Nutrition and diet. *Asian Small Clawed Otter Husbandry Manual*.

[27] Davison R. P., Mautz W. W., Hayes H. H., Holter J. B. (1978). The efficiency of food utilization and energy requirements of captive female Fishers. *The Journal of Wildlife Management*.

[28] Powers J. G., Mautz W. W., Pekins P. J. (1989). Nutrient and energy assimilation of prey by bobcats. *The Journal of Wildlife Management*.

[29] Earle K. E., Smith P. M. (1991). Digestible energy requirements of adult cats at maintenance. *The Journal of Nutrition*.

[30] Davis H. G., Aulerich R. J., Bursian S. J., Sikarskie J. G., Stuht J. N. (1992). Feed consumption and food transit time in northern river otters (*Lutra canadensis*). *Journal of Zoo and Wildlife Medicine*.

[31] Melissen A., Nijboer J., Hatt J. M., Kaumanns W., Bejnen A., Ganslober U. (2000). Variation in energy intake in Eurasian otters (*Lutra lutra*): effects of lactation and seasonal changes. *Zoo Animal Nutrition*.

[32] Ruff K. A. (2007). *Optimizing the Nutrition of Captive Eurasian Otters (Lutra lutra). Ph.D. Thesis.*.

[33] Vester B. M., Beloshapka A. N., Middelbos I. S. (2010). Evaluation of nutrient digestibility and fecal characteristics of exotic felids fed horse or beef based diets: use of the domestic cat as a model for exotic felids. *Zoo Biology*.

[34] Kerr K. R., Morris C. L., Burke S. L., Swanson K. S. (2013). Apparent total tract macronutrient and energy digestibility of 1- to- 3-day-old whole chicks, adult ground chicken, and extruded and canned chicken-based diets in African wildcats (*Felis silvestris* lybica). *Zoo Biology*.

[35] Kerr K. R., Morris C. L., Burke S. L., Swanson K. S. (2014). Apparent total tract energy and macronutrient digestibility of one- to three-day-old, adult ground, extruded, and canned chicken-based diets in domestic cats (*Felis silvestris catus*). *Journal of Animal Science*.

[36] Warner A. C. I. (1981). Rate of passage of digesta through the gut of mammals and birds. *Nutrition Abstracts and Reviews B*.

[37] Trout D. L., Conway E. S., Putney J. D. (1977). Dietary influences on gastric emptying of carbohydrate versus fat in the rat. *The Journal of Nutrition*.

[38] Porsgaard T., Straarup E. M., Høy C.-E. (2003). Gastric emptying in rats following administration of a range of different fats measured as acetaminophen concentration in plasma. *Annals of Nutrition and Metabolism*.

[39] Fisher K. I., Stewart R. E. A., Kastelein R. A., Campbell L. D. (1992). Apparent digestive efficiency in walruses (*Odobenus rosmarus*) fed herring (*Clupea harengus*) and clams (*Spinsula* sp.). *Canadian Journal of Zoology*.

[40] Kienzle E., Stratmann B., Meyer H. (1991). Investigations on palatability, digestibility and tolerance of low digestible food components in cats. *The Journal of Nutrition*.

[41] Ahlstrom Ø., Skrede A. (1995). Comparative nutrient digestibility in blue foxes (*Alopex lagopus*) and mink (Mustela vison) fed diets with diverging fat: carbohydrate ratios. *Acta Agriculturae Scandinavica, Section A—Animal Science*.

[42] Kienzle E., Dobenecker B., Eber S. (2001). Effect of cellulose on the digestibility of high starch versus high fat diets in dogs. *Journal of Animal Physiology and Animal Nutrition*.

[43] Romsos D. R., Belo P. S., Bennink M. R., Bergen W. G., Leveille G. A. (1976). Effects of dietary carbohydrate, fat and protein on growth, body composition and blood metabolite levels in the dog. *The Journal of Nutrition*.

[44] Geng Y. Y., Yang F. H., Xing X. M., Gao X. H. (2012). Effects of dietary fat levels on nutrient digestibility and production performance of growing-furring blue foxes (*Alopex lagopus*). *Journal of Animal Physiology and Animal Nutrition*.

[45] Eggum B. O., Thorbek G., Beames R. M., Chwalibog A., Henckel S. (1982). Influence of diet and microbial activity in the digestive tract on digestibility, and nitrogen and energy metabolism in rats and pigs. *British Journal of Nutrition*.

